# Characterizing the *Staphylococcus aureus* fatty acid degradation operon

**DOI:** 10.1128/jb.00089-25

**Published:** 2025-07-17

**Authors:** Cindy Menjivar, Zachary R. DeMars, Richard E. Wiemels, Ronan K. Carroll, Jeffrey L. Bose

**Affiliations:** 1 Department of Microbiology, Molecular Genetics, and Immunology, University of Kansas Medical Center 21638, Kansas City, Kansas, USA; 2 Department of Biological Sciences, Ohio University 110002 , Athens, Ohio, USA; The Ohio State University, Columbus, Ohio, USA

**Keywords:** MRSA, fatty acid, beta-oxidation, CcpA, FadB

## Abstract

*Staphylococcus aureus* can supplement its endogenous fatty acid synthesis pathway (FASII) with exogenous fatty acids it acquires from the environment through the fatty acid kinase (Fak) complex. Although *S. aureus* has been thought to not degrade fatty acids, it does possess a potential *fadXDEBA* locus that contains all the genes necessary for β-oxidation. Using mRNA analysis, we determined that the *fadXDEBA* operon can be found on one polycistronic mRNA. Moreover, we identified the *fadX* promoter and a putative binding site within this region that is consistent with negative regulation by the metabolism-responsive regulator, Carbon Catabolite Protein A (CcpA). Indeed, in the absence of glucose or CcpA, we saw the *fadXDEBA* operon was derepressed. *S. aureus* is annotated to lack the crotonase domain of FadB; however, new analysis indicates it is present. To test the functionality of the *S. aureus* FadB, we performed complementation assays with *E. coli fad* mutants using minimal media supplemented with single fatty acids. We were able to restore the growth of *E. coli fad* mutants when providing *safadBA* genes on a plasmid and demonstrate that the SaFadB crotonase domain is required for complementation. Together, these data demonstrate the SaFadBA proteins are functional within a well-characterized fatty acid degradation system, and the *S. aureus fadXDEBA* operon is under strong catabolite repression.

**IMPORTANCE:**

*Staphylococcus aureus* has long been thought to lack a functional fatty acid degradation (Fad) pathway based on limited studies. Pathway analysis suggested the *S. aureus* FadB protein lacks a crotonase domain, which is essential for Fad activity. This study demonstrates that *S. aureus* FadB possesses a crotonase domain that has eluded identification likely due to the orientation of its two enzymatic domains. In addition, we show that the Fad pathway is under strong catabolite repression under standard laboratory conditions, which may have also contributed to its lack of detected activity. A new model of fatty acid metabolism is emerging in *S. aureus* that changes the understanding of how this bacterium synthesizes and metabolizes fatty acids.

## INTRODUCTION

*Staphylococcus aureus* is a successful gram-positive human pathogen with the ability to cause infection at almost any anatomical site resulting in a wide range of diseases (1–5). The capability of *S. aureus* to establish high-morbidity infections can be attributed to its diverse repertoire of virulence factors that range from secreted toxins to immune evasion proteins (6, 7) and a versatile metabolism that allows it to survive in a variety of niches (8–12). Like most bacteria, *S. aureus* is equipped with an intricate sensing regulatory network that responds to cellular, host, and environmental cues, allowing for its adaptability.

Fatty acids are an essential component for all life forms, and exogenous fatty acids (exoFAs) can be found in most environments, including the skin and host tissue, where they contact human pathogens. Bacteria can use host fatty acids for different purposes, including the synthesis of bacterial lipids, as components of signaling molecules, for membrane homeostasis, or as a carbon source through β-oxidation (13, 14). *S. aureus*, like most bacteria, not only possesses an endogenous fatty acid biosynthesis (FASII) pathway that allows it to synthesize fatty acids, but can also acquire exoFAs from its environment. Previously, we have shown that *S. aureus* can incorporate exogenous unsaturated fatty acids from host tissue via the fatty acid kinase (Fak) system and determined the importance of this system in maintaining membrane integrity (13). Although one fate of exoFAs could be direct incorporation into the phospholipid bilayer, many bacteria can break down fatty acids using a fatty acid degradation (Fad) pathway, also known as β-oxidation (14).

Although *S. aureus* possesses several major metabolic pathways, it is thought to not possess a full Fad pathway (15, 16). Indeed, a previous study failed to detect fatty acid degradation in *S. aureus* strain RN4220 after incubation with [1-^14^C]18:1Δ9 yielded only fatty acid species, leaving the Fatty Acid Kinase (FakA), the only known way in which exoFAs can be utilized (16). This notion has been widely accepted until recently, when the first significant study on *S. aureus* Fad demonstrated that the first step of this pathway can occur (17). The canonical Fad pathway consists of four enzymatic proteins that work in a cyclic manner to degrade exoFAs by two carbons every cycle (18). First, exoFAs are activated by the acyl-CoA synthetase (FadD) with unsaturation of the acyl chain occurring by the acyl-CoA dehydrogenase (FadE). Subsequently, oxidation and hydration occur by the hydroxyacyl-CoA dehydrogenase/enoyl-CoA hydratase (FadB). Finally, the ketothiolase (FadA) cleaves the fatty acid and thus generates the two-carbon metabolite acetyl-CoA. Annotations of *S. aureus* genomes readily identify the proteins FadD, FadE, and FadA, but the bifunctional protein FadB has been largely misannotated as lacking the essential enoyl-CoA hydratase (crotonase) domain, contributing to the dogma that *S. aureus* lacks a functional Fad pathway.

Metabolic flux in *S. aureus* has been immensely studied, particularly with the involvement of a preferred carbon source such as glucose (19–21). The cellular response to glucose availability leads to the repression of the TCA cycle and secondary metabolism by the Catabolite Control Protein A (CcpA), a highly conserved transcriptional regulator in low-GC-gram-positive bacteria (22–24). Its involvement in controlling carbon-metabolism pathways lies in its carbon catabolite repression (CCR) and binding to DNA promoters and some RNAs (25), where CCR allows bacteria to modulate the expression of specific pathways in response to preferred nutrient availability (24, 26, 27). Previously, we identified decreased CcpA activity in a Δ*fakA* mutant at 3 h but increased at later time points, linking exoFA metabolism with central metabolic processes (28).

Fatty acid metabolism has been a point of interest for the generation of new therapeutics that target the FASII system (11, 29, 30). The effectiveness of such compounds depends on how bacteria metabolize fatty acids. In *S. aureus*, the importance of FASII and FakA has been the subject of investigation; however, the Fad pathway has yet to be extensively studied. In this current study, we examine the genetic composition of the *fadXDEBA* locus as well as demonstrate that it is derepressed when CcpA is absent or when preferred carbon sources are omitted. In addition, we tested the ability of the *S. aureus* FadBA proteins to function within a well-characterized Fad system and identified the previously overlooked crotonase domain of FadB, supporting our hypothesis that the *S. aureus* Fad pathway is complete. Our studies, along with others, provide new insights into fatty acid metabolism in *S. aureus* and provide a revised model for how *S. aureus* metabolizes fatty acids.

## RESULTS

### *S. aureus* possesses a locus encoding a putative complete fatty acid degradation pathway

**Fig 1:**
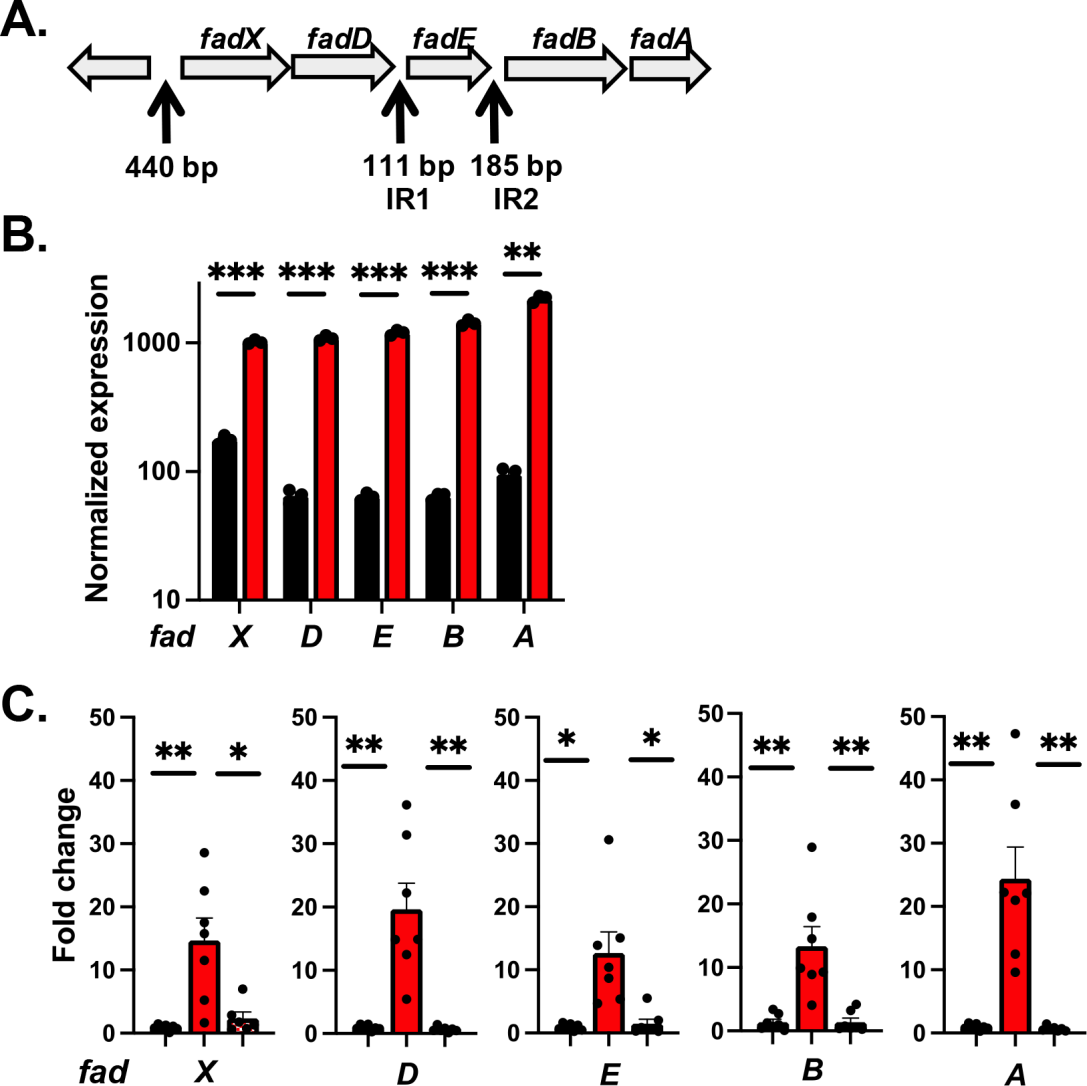
The *fad* genes are upregulated in a *fakA* mutant. (**A**) Schematic of the *fadXDEBA* operon in *S. aure*us. Therefore, “440 bp” is the spacing between *fadX* and the upstream gene and contains the promoter. Intergenic regions (IR1 and IR2) include size (bp). (**B**) Expression of *fad* genes using RNA-seq in wild-type (black bars) and Δ*fakA* mutant (red bars) grown to 6 h in TSB supplemented with 14 mM glucose. Data are the mean (*n* = 3) with SEM. (**C**) qRT-PCR of *fad* genes in wild-type (black bars), Δ*fakA* (red bars)*,* Δ*fakA* with *fakA* expressed on a plasmid (red and white hatched bars) grown to 6 h in TSB supplemented with 14 mM glucose. Data are the mean (*n* = 6 or 7) with SEM. * Indicates *P* < 0.05, ** indicates *P* < 0.01, *** indicates *P* < 0.001 by student’s *t*-test, with lines indicating comparisons.

Previously, we observed that in tryptic soy broth (TSB) supplemented with 14 mM glucose, a Δ*fakA* mutant had an enhanced late-exponential-phase growth and altered metabolism compared with wild-type. We observed changes in key metabolites, an altered acetate switch, and a difference in the redox state in the cell (28). In addition, we identified both increased and decreased activity of CcpA, a regulator of metabolic function, at different time points (31). To gain a larger view of gene expression changes responding in the absence of FakA, we performed RNA-seq from samples collected under those same conditions (TSB supplemented with 14 mM glucose) on a wild-type and a Δ*fakA* mutant at the late exponential phase of growth (6 h). Although we observed a variety of changes in the Δ*fakA* mutant, the locus with the largest fold expression increase was a putative *fadXDEBA* locus (Fig. 1A) with an average of 17-fold higher expression (Fig. 1B). Unlike in the Δ*fakA* mutant, these genes were poorly expressed in the wild-type strain. To corroborate these results, we performed real-time qPCR on the putative *fad* genes and saw the same higher expression in the Δ*fakA* mutant, which could be restored to wild-type levels when *fakA* was reintroduced on a plasmid (Fig. 1C).

**Fig 2:**
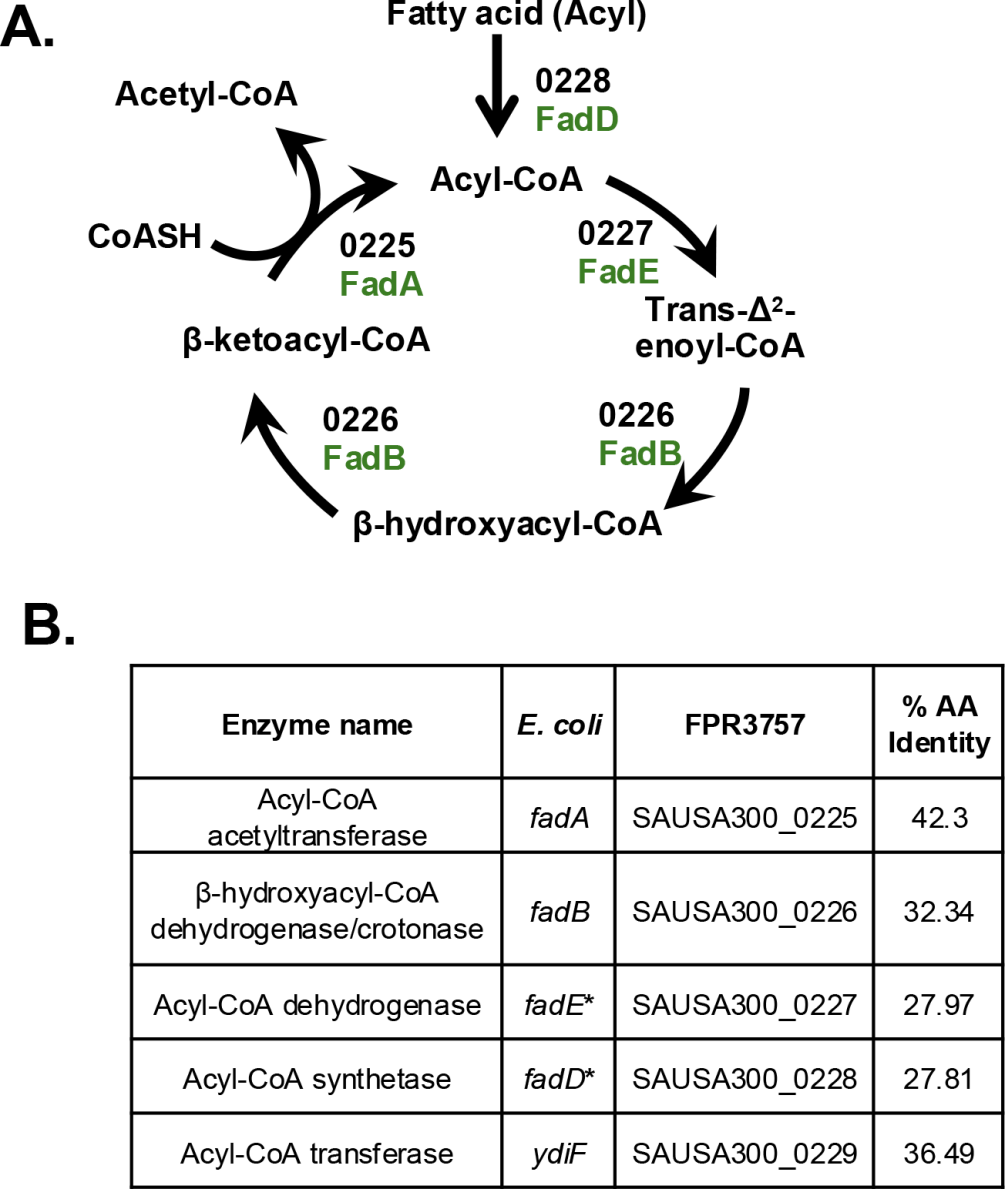
*S. aureus* encodes a Fad pathway. (**A**) Schematic of a standard Fad pathway with *E. coli* protein designations are labeled in green, and putative *S. aureus* ORF (SAUSA300_XXXX) numbers are labeled in black. The cycle decreases the fatty acid length by two carbons per cycle as acetyl-CoA is released. (**B**) Table of putative functions and gene designations based on annotation and analysis from studies in *E. coli*. The final column indicates the % amino acid identity between the *S. aureus* protein and those found in *E. coli*. * Indicates mis-annotation of genes in *S. aureus* genome; FadE and FadD are swapped.

Although *S. aureus* has been thought to not possess a functional Fad pathway, the *fad* locus does appear to encode the proteins necessary for a complete pathway (Fig. 2A). This is supported by a recent study suggesting as much and demonstrating the activity of the first step, performed by FadD, does occur (17). Our bioinformatic analysis agrees with their data and suggests a canonical Fad pathway with an accessory protein, SAUSA300_0229, which is annotated as FadX and is a putative acyl-CoA transferase. FadX is predicted to be analogous to YdiF in *Escherichia coli* (Fig. 2B). It should be noted that the FPR3757 genome appears to be misannotated for SAUSA300_0228 and SAUSA300_0227 compared with conventional designations based on function. The former is labeled as *fadE* and is predicted to encode an acyl-CoA synthetase, which is known as FadD in *E. coli*. The latter is annotated as *fadD* and predicted to be an acyl-CoA dehydrogenase, which is FadE in *E. coli*. Our bioinformatic predictions agree with the functional name and not the *S. aureus* gene designations. This was also noted by another group (17), and we will therefore use these updated designations that are based on predicted function as seen in Fig. 2B. When referring to the *S. aureus* protein, we will add “Sa” to the name, such as SaFadB. Together, these data predict that *S. aureus* encodes all the proteins necessary for fatty acid degradation.

### The genetic content of the *fad* locus

**Fig 3:**
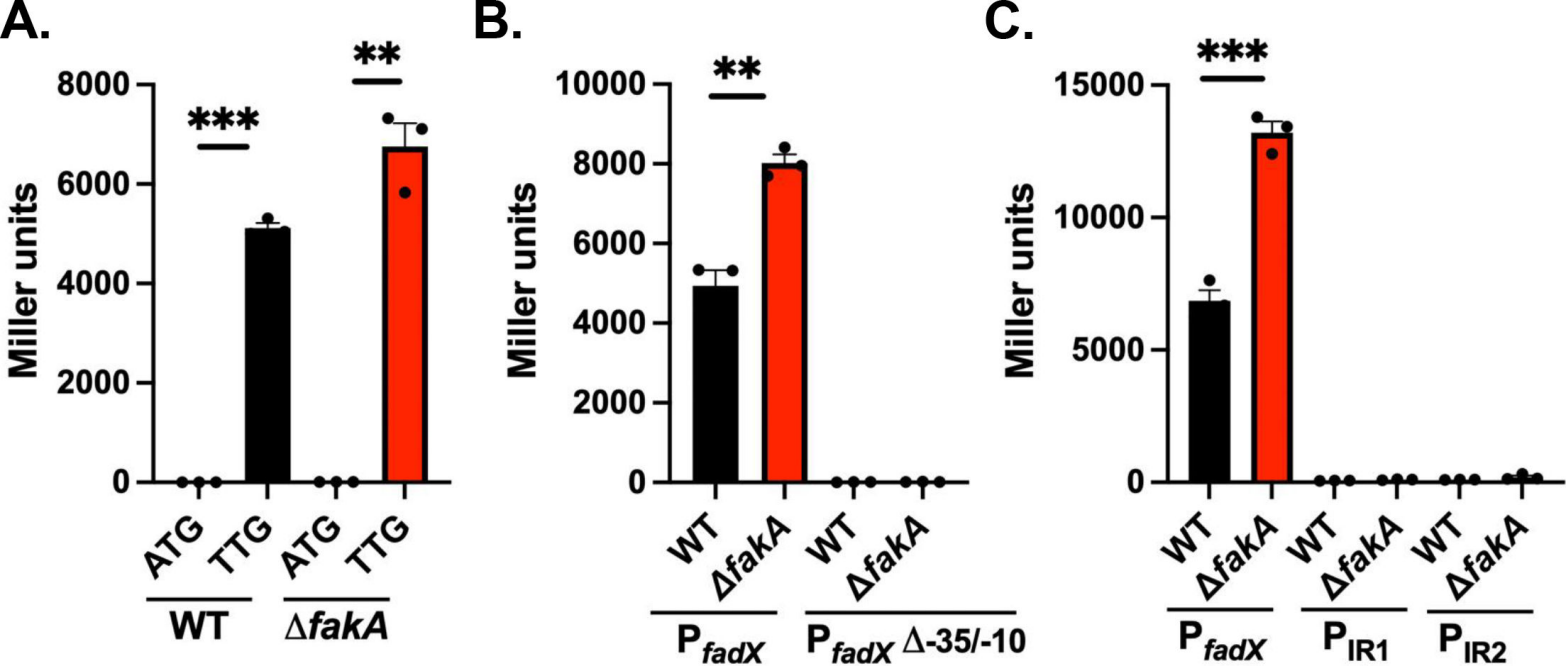
Characterization of the *fad* regulatory elements. (**A**) Wild-type (WT) strain and Δ*fakA* mutant harboring β-galactosidase reporter plasmids for putative “ATG” and “TTG” translational start sites (P*_fadX_* = “TTG” reporter). (**B**) Strains harboring P*_fadX_* and deletion of putative -35/-10 β-galactosidase reporter plasmid. (**C**) Strains harboring β-galactosidase reporter plasmids for P*_fadX_* or the two intergenic regions (P_IR1_ and P_IR2_). All assays were performed with samples collected at 6 h in TSB supplemented with 14 mM glucose. Bars represent the mean (*n* = 3) with SEM. ** Indicates *P* < 0.01, *** indicates *P* < 0.001 by student’s *t*-test with lines indicating comparisons.

Little is known about the putative *fad* locus; therefore, we sought to characterize its genetic composition. During our initial examination, we noted that the annotated *fadX* start codon was an ATG but possessed no identifiable Shine-Dalgarno site; however, 13 nucleotides downstream is a TTG with a well-spaced and almost canonical Shine-Dalgarno sequence (Fig. S1A). The use of alternative start codons such as TTG is not atypical in *S. aureus* (32–34). This led us to consider whether the start site of *fadX* was incorrectly annotated. To test which of the two putative codons is correct, we generated β-galactosidase translational reporter plasmids for the two start sites with the native promoter and examined expression in our wild-type and Δ*fakA* mutant strains. We observed expression only for the “TTG” reporter in both strains (Fig. 3A). Consistent with our RNA analysis, this reporter was also more highly expressed in the Δ*fakA* mutant compared with the wild-type. To verify whether the “ATG” reporter had no activity, we extended the β-galactosidase assay incubation period overnight and were still unable to detect any expression (data not shown). These data demonstrate that the “TTG” is the translational start site for *fadX,* and the “ATG” is a misannotation in the genome.

We noted a putative promoter site with high homology (4/6 for −35 and 5/6 for −10 with 16 spaces between) to consensus σ^A^ −35 and −10 sequences (Fig. S1A) within our cloned *fadX* promoter region. We hypothesized that if these sequences are the promoter elements, deleting them will abolish the expression. Indeed, a β-galactosidase translational reporter (P*_fadX_*_Δ*-35/-10*_) without this sequence had no expression in either wild-type or the Δ*fakA* mutant (Fig. 3B), demonstrating that this region is essential for *fadX* expression and is a critical promoter element.

The *fadXDEBA* genes are present in a single locus and responded similarly in a Δ*fakA* mutant during our mRNA analysis (Fig. 1B and C), suggesting that they are expressed in a single operon. However, there is significant spacing between the *fadD* and *fadE* genes (111 base pairs, intergenic region 1, IR1) and between the *fadE* and *fadB* genes (185 base pairs, IR2) that could contain promoters (Fig. 1A). To test this, we constructed β-galactosidase reporter plasmids for the two intergenic regions (Fig. S1B) and compared the two new reporters with the P*_fadX_-lacZ* reporter. Since we saw high *fad* expression in a Δ*fakA* mutant during our mRNA analysis, we tested these reporters in both wild-type and Δ*fakA* mutant under the same conditions (6 h, TSB with glucose). Although P*_fadX_* activity was easily detected, no expression was observed from P_IR1_ or P_IR2_, suggesting that promoters are absent in the intergenic regions or, if there are promoters, they are not active under these conditions (Fig. 3C). Furthermore, we analyzed the RNA-seq data sets from 44 different conditions in *S. aureus* strain HG001 (35). An analysis of their results demonstrates that all *fad* genes are expressed at similar levels in all conditions tested, further indicating that they are expressed on a single transcript (Fig. S2).

**Fig 4:**
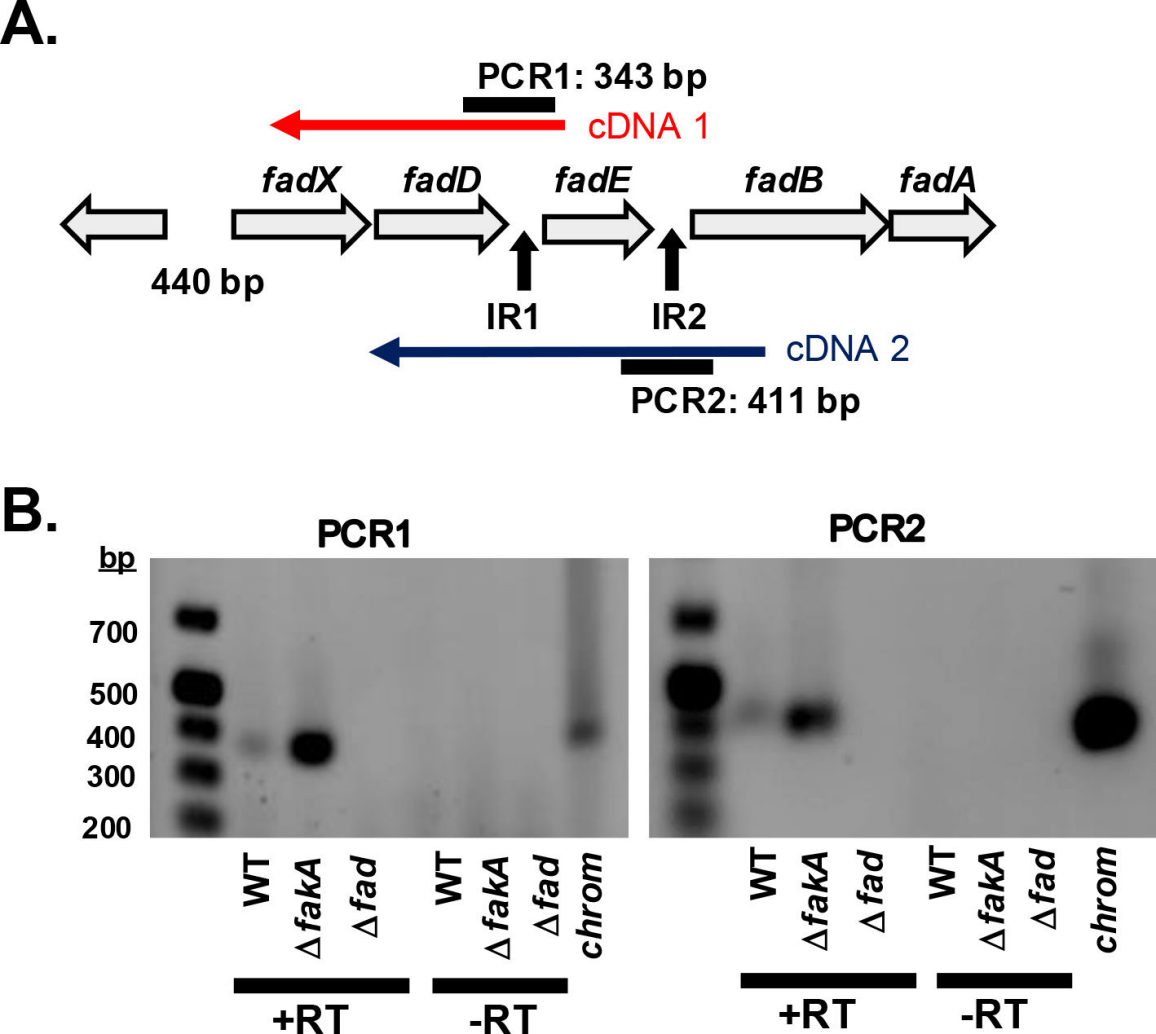
The *fad* mRNA extends across intergenic regions. (**A**) Schematic of *fadXDEBA* operon with an indication of cDNA synthesis generation and PCR products amplifying the intergenic regions (IR1 and IR2). (**B**) Reverse-transcriptase PCR from RNA extracted from WT, Δ*fakA,* and Δ*fadXDEBA* (Δ*fad*) mutants from 6 h cultures grown in TSB with 14 mM glucose. Full gel images are available in Fig. S9.

To confirm that *fadXDEBA* genes can be encoded on a single mRNA, we performed reverse-transcriptase PCR with our wild-type, Δ*fakA* mutant, and a Δ*fadXDEBA* (Δ*fad*) deletion strains. cDNA was generated with primers internal to *fadE* and *fadB,* and we then amplified across the intergenic regions (Fig. 4A). We included the Δ*fakA* mutant again since the *fad* locus is poorly expressed in wild-type but should be readily detectable in the absence of FakA. A PCR product was obtained by both reactions (Fig. 4B), demonstrating that mRNA spans the intergenic regions and is consistent with *fadXDEBA* being a polycistronic mRNA. Again, each PCR reaction yielded a stronger product in the Δ*fakA* mutant compared with wild-type.

### The *fadXDEBA* promoter is under carbon catabolite repression

Recently, Kuiack et al. demonstrated using a P*_fadX_-lux* reporter in their USA300 *S. aureus* strain that the highest expression of *fad* occurred during the stationary phase (17). Moreover, they observed increased expression in the presence of fatty acid, palmitic acid, and the absence of glucose. Although we observed higher expression of the P*_fadX_-lacZ* reporter during the transition from exponential to stationary phase (8 h), we did not observe an increase upon palmitic acid addition (Fig. S3A). Again, no significant expression was observed from the P_IR1_ and P_IR2_ reporters. Of note, all strains grew similarly under all growth conditions tested (Fig. S4).

**Fig 5:**
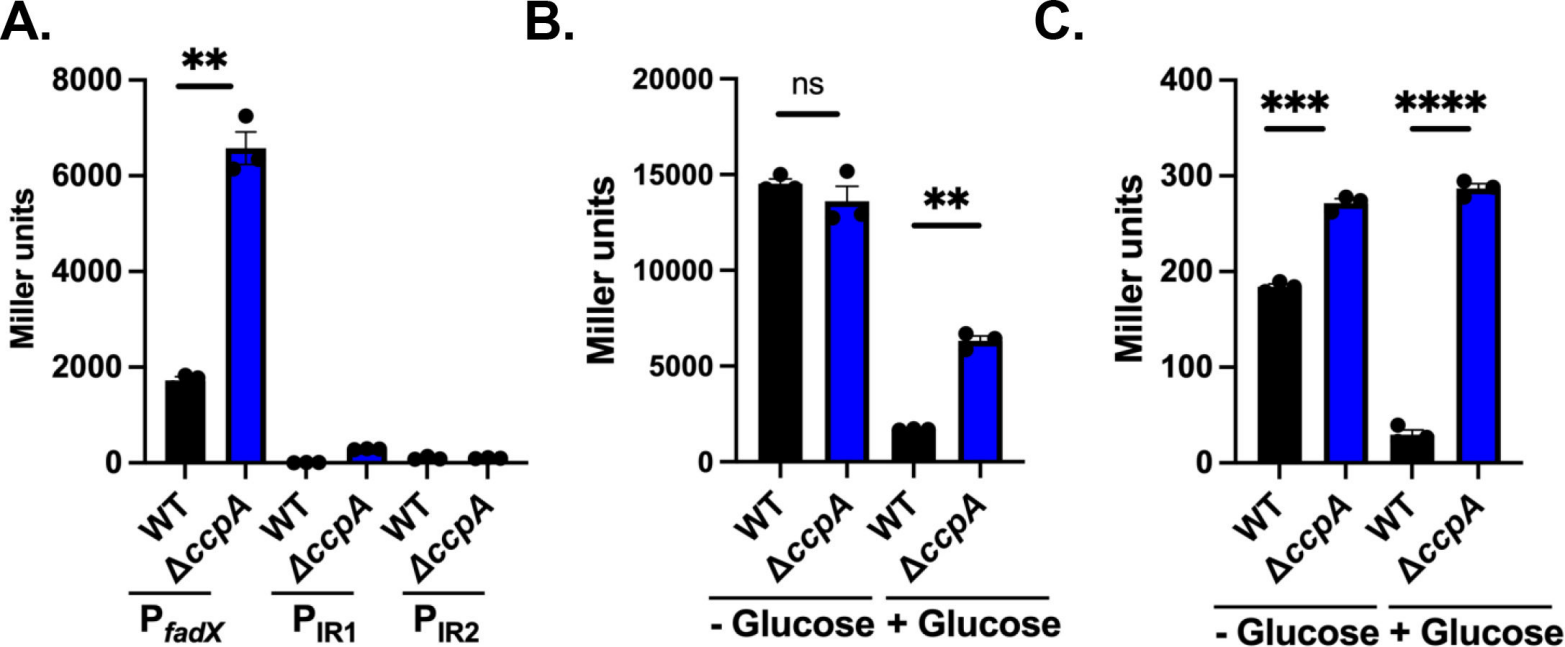
*fad* is derepressed in the absence of CcpA. (**A**) β-galactosidase assay with wild-type strain and Δ*ccpA* mutant harboring P*_fadX_*, P_IR1_, or P_IR2_ reporters grown for 4 h in TSB supplemented with 14 mM glucose. (**B**) β-galactosidase assay with wild-type strain and Δ*ccpA* mutant harboring the P*_fadX_* reporter plasmid and grown to 4 h in TSB with or without 14 mM glucose. (**C**) β-galactosidase assay with wild-type strain and Δ*ccpA* mutant harboring P_IR1_-*lacZ* reporter grown for 4 h in TSB with or without 14 mM glucose. Bars represent the mean (*n* = 3) with SEM. “ns” indicates no significant difference, ** indicates *P* < 0.01, *** indicates *P* < 0.001, and **** indicates *P* < 0.0001 by Student’s *t*-test, and lines indicate comparisons.

The absence of glucose led to a significant induction of P*_fadX_-lux* expression in the previous study, although the mechanism by which this occurs was not determined. However, it agrees with our RNA-seq data, demonstrating low expression in our wild-type strain in TSB with glucose (Fig. 1) and suggests glucose can influence the expression of the *fadXDEBA* operon. *S. aureus*, like many bacteria, often suppresses secondary metabolic processes when a preferred carbon source, such as glucose, is present (26, 27, 36). In gram-positive bacteria, this is largely controlled by Carbon Control Protein A (CcpA). A careful analysis of the *fadX* promoter revealed a putative *cre* site (TTGTAAGGGTTTACAC, underlined non-consensus, Fig. S5), the binding site for CcpA (37, 38), downstream of the putative −10 sequence (Fig. S1A). This site has been previously noted as a potential *CRE* site in the *S. aureus* N315 genome on the RegPrecise website (39). Based on this, we hypothesized that the *fad* operon is under CcpA repression. To test this, we evaluated the activities of P*_fadX_*, P_IR1_, and P_IR2_-*lacZ* reporters in a Δ*ccpA* mutant grown in TSB supplemented with 14 mM glucose at 4 h. This time point was chosen since it is before glucose exhaustion (19, 28). Consistent with CcpA being a repressor of *fad* expression, we observed higher expression of P*_fadX_* when CcpA was absent (Fig. 5A). Again, we saw no expression from the P_IR2_ reporter; however, we identified a slight increase in P_IR1_ expression in the Δ*ccpA* mutant (Fig. 5A). Of note, Kuiack et al. previously noted a potential *cre* site in IR1 (Fig. S5B) but did not test its importance (17). Since CcpA is active when glucose is present, we reasoned that the absence of glucose should eliminate the dependency on *fad* expression by CcpA. To test this, we grew our wild-type and Δ*ccpA* mutant strains containing the P*_fadX_* reporter in the presence and absence of glucose. Again, we observed increased expression when CcpA was absent while glucose was present; however, no difference was observed between wild-type and Δ*ccpA* mutants in the absence of glucose, further supporting CcpA as a repressor of *fad* expression (Fig. 5B). To corroborate the results of the P_IR1_-*lacZ* reporter that contains the second *cre* site, we grew it in the presence and absence of glucose, where we observed higher expression in the Δ*ccpA* mutant compared with wild-type in both conditions. We also observed higher expression in the wild-type in the absence of glucose (Fig. 5C). CcpA responds to carbon sources that generate fructose-1,6-bisphosphate produced during glycolysis. We tested whether fructose, which also generates this glycolysis intermediate, would similarly inhibit P*_fadX_* expression at 4 h. Indeed, fructose inhibited reporter expression similar to that of glucose (Fig. S3B).

We previously demonstrated that a Δ*fakA* mutant has altered *gltA* expression, a CcpA-dependent mRNA (28). This expression was intermediate between wild-type and Δ*ccpA* mutant. FakA is a kinase of fatty acids with no apparent ability to regulate promoters directly; therefore, we hypothesized that our observation of increased *fad* expression in the Δ*fakA* mutant is the result of its impact on CcpA activity. To determine this, we examined the expression of the P*_fadX_* reporter under several growth conditions. We previously demonstrated that under our growth conditions, glucose is present at 4 h but is exhausted by 6 h (28). Thus, these time points represent times during growth when CcpA would be active and inactive, respectively. Based on this, we tested our P*_fadX_* reporter in our wild-type, Δ*fakA,* Δ*ccpA*, and Δ*fakA*Δ*ccpA* double mutants at 4 (Fig. S3C) and 6 (Fig. S3D) h of growth. Consistent with the previously seen CcpA repression, we observed enhanced promoter activity in the Δ*ccpA* and Δ*fakA*Δ*ccpA* mutants at 4 h of growth compared with wild-type. At 6 h, all four strains demonstrated increased expression, with the Δ*fakA* mutant reaching expression levels similar to the Δ*ccpA* mutant and Δ*fakA*Δ*ccpA* double mutant (Fig. S3D). Moreover, since the Δ*fakA*Δ*ccpA* double mutant mirrored the single Δ*ccpA* mutant, we interpreted this as the enhanced expression of *fad* in the Δ*fakA* mutant is due to changes in CcpA activity. Together, these results demonstrate that *fad* is under strong catabolite repression in the presence of a preferred carbon source and is derepressed when CcpA is absent.

### The putative SaFadBA proteins can complement the *E. coli* fatty acid degradation system

**Fig 6:**
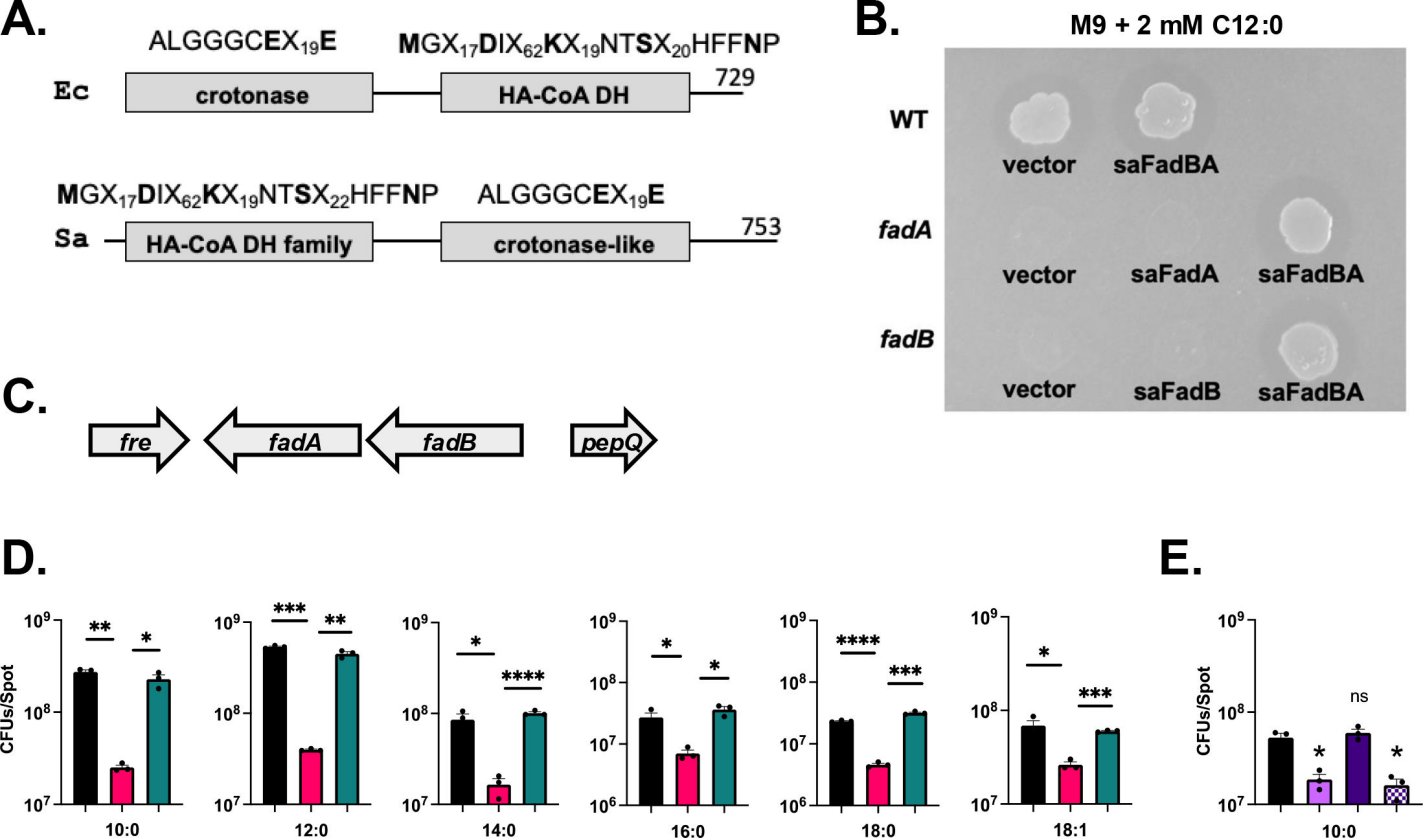
*S. aureus* has a functional FadB. (**A**) Domain architecture of *E. coli* (Ec) and *S. aureus* (Sa) FadB showing crotonase and β-hydroxyacyl-CoA dehydrogenase (HA-CoA DH) domains. Key amino acids for function are shown in “**bold,**” based on data from UniProt. ”X” indicates any amino acid. The total number of amino acids is indicated by the number on the right. (**B**) Growth of indicated strains on minimal media containing 2 mM lauric acid (C12:0), 0.1 mM IPTG, and 100 μg mL^−1^ ampicillin. Top row: MG1655 *E. coli* wild-type (WT) harboring empty vector or SaFadBA. Middle row: *fadA*::Tn10 harboring empty vector or SaFadBA and Δ*fadA* harboring plasmid expressing SaFadA. Bottom row: Δ*fadB* mutant harboring empty vector or a plasmid expressing SaFadB or SaFadBA. The image is representative of several independent experiments. (**C**) Diagram of *fadBA* operon orientation in *E. coli*. (**D**) Equal numbers of *E. coli* MG1655 wild-type (WT) (black bar), *fadA*::Tn10 mutant (pink bar), and mutant harboring plasmid expressing SaFadBA (teal bar) were grown on minimal media plates containing 2 mM fatty acids C10:0 C18:1. Bacterial numbers were determined after 7 days. (**E**) Equal numbers of *E. coli* BW25113 wild-type (WT) (black bar), Δ*fadB* mutant (lavender bar), and mutant harboring plasmids expressing SaFadBA (purple bar) or SaFadBA* (E^550^A and E^570^A in *fadB*) (purple and white hatched bar) were grown on minimal media plates containing 2 mM C10:0. After 7 days, bacterial numbers were determined. The bars represent the mean with SEM (*n* = 3). * Indicates *P* < 0.05, ** indicates *P* < 0.01, *** indicates *P* < 0.001, **** indicates *P* < 0.0001 by Student’s *t*-test with lines indicating comparisons. In panel E, significance is compared with wild-type.

One reason *S. aureus* has been thought to lack a functional Fad pathway is due to a previous group’s inability to detect acyl-CoA molecules in a *S. aureus* RN4220 strain, although the growth conditions and methods were not listed and data were not shown (15). Conversion of palmitic acid to palmitoyl-CoA has now been demonstrated (17). Another reason contributing to this dogma is that both BioCyc and KEGG pathway analysis reveal that *S. aureus* lacks the crotonase activity of the bifunctional FadB, which typically has both acyl-CoA dehydrogenase and crotonase activity. Indeed, SAUSA300_0226 is annotated as a 3-hydroxyacyl-CoA dehydrogenase only. Thus, the most obvious missing Fad activity is the essential crotonase. To examine this closer, we performed structural predictions using Phyre2 (40) on the amino acid sequence of the *S. aureus* FadB protein and were able to model the N-terminal domain to be a 3-hydroxyacyl-CoA dehydrogenase, which is the C-terminal domain of *E. coli* FadB. However, examination of the SaFadB C-terminal domain alone matched the *E. coli* FadB crotonase domain (Fig. 6A). Both domains possess residues identified to be important for their respective enzymatic activities (41). Thus, we predict the SaFadB to have both functional FadB domains with the exception that the domains are in opposite orientations, which could have led to the lack of identification.

To support our bioinformatic analysis, we sought to demonstrate the functionality of SaFadB protein by taking advantage of *E. coli fad* mutants, a successful strategy previously used with FadBA proteins of *Pseudomonas fragi*, *Mycobacterium tuberculosis*, and *Streptomyces coelicolor* (42–44). To this end, we cloned *safadA*, *safadB*, and both genes together into an *E. coli* expression plasmid and examined their ability to complement the ability of *E. coli fad* mutants to grow with fatty acids as a sole carbon source. All strains were able to grow on M9 supplemented with glucose as expected (Fig. S6A). SaFadB and SaFadA alone were unable to restore growth of the *E.* coli ΔfadB, Δ*fadA*, and *fadA*::Tn10 mutants, respectively (Fig. 6B). The *fadA* and *fadB* genes are found on a single operon in *E. coli* (Fig. 6C), and the two proteins form a tetramer consisting of two monomers of each protein in other bacteria including *E. coli* and *P. fragi* (41, 44, 45). We suspected the sequence differences between species, or the domain orientation, could account for the failure of the single-gene complementation. Based on this, we co-expressed SaFadBA and were able to rescue the growth of the *E.* coli Δ*fadB* and *fadA*::Tn10 mutant (Fig. 6B). Furthermore, we were able to quantify this complementation on a variety of fatty acids (Fig. 6D).

Using the AlphaFold model of SaFadB N- and C-terminal domains, we modeled their predicted structures onto the *E. coli* FadB protein (Fig. S7A). This approach demonstrates the conservation of the crotonase active site with the essential glutamic acid residues (E550 and E570) necessary for enzymatic activity (Fig. 6A; Fig. S7B) (41). To further demonstrate that SaFadB does indeed encode a crotonase domain, we hypothesized that mutation of E550 and E570 to alanine would render the SaFadB nonfunctional. To this end, we generated both mutations in our SaFadBA plasmid and attempted to complement the *E.* coli Δ*fadB* mutant on a single fatty acid. Mutation of these amino acids abolished the ability of the co-expressed SaFadBA to restore growth (Fig. 6E; Fig. S6B). Together, these data demonstrate for the first time that the *S. aureus* FadBA proteins are functional within a well-characterized fatty acid degradation system and that the SaFadB protein does possess the functions of a canonical FadB.

## DISCUSSION

**Fig 7:**
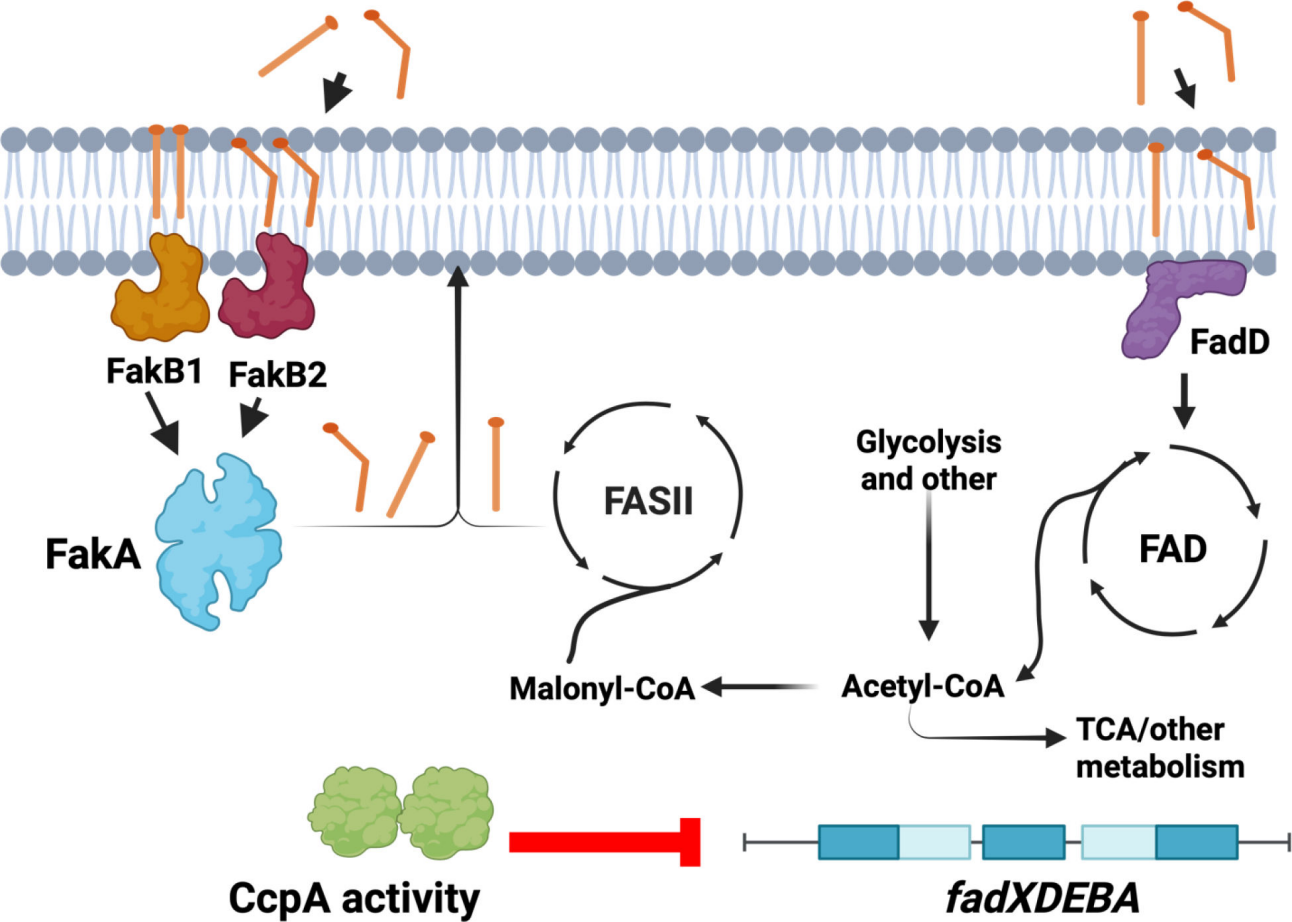
Model for fatty acid metabolism in *S. aureus*. The bacterium can synthesize fatty acids through the fatty acid biosynthetic pathway (Fab, FASII) from malonyl-CoA. In addition, *S. aureus* can supplement fatty acid production for lipid synthesis with the use of exogenous fatty acids through the fatty acid kinase (Fak) complex that consists of two fatty acid binding proteins (FakB1 and FakB2) and FakA. These fatty acids can then be incorporated into the phospholipid bilayer. Alternatively, exogenous fatty acids are processed through fatty acid degradation (FAD), where the generation of acetyl-CoA could also supplement FASII. CcpA represses (red line) the expression of secondary metabolic processes such as the *fadXDEBA* operon. Produced using bioRender.

A model of fatty acid metabolism is emerging in *S. aureus* that changes the understanding of how this bacterium synthesizes and metabolizes fatty acids (Fig. 7). Like many bacteria, *S. aureus* can produce fatty acids endogenously using the FASII pathway, and these endogenously produced fatty acids are used to make phospholipids, glycolipids, as well as anchors for membrane-associated molecules (46, 47). In addition, *S. aureus* can supplement this endogenous system with exoFA lipid integration via the fatty acid kinase complex, but additional fates for these exoFAs are uncertain. Indeed, *S. aureus* has long been thought to not possess a Fad pathway; however, this has been challenged by recent studies, and in this study, we provide further evidence that *S. aureus* has a fatty acid degradation pathway. Moreover, we demonstrate that it is under strong catabolite repression in the presence of a preferred carbon source.

A contributing factor to the thought that *S. aureus* lacks a full Fad pathway is a bioinformatic analysis that predicted most of the Fad activities are present, but not all. Although FadD and FadE are annotated, as noted by the McGavin group and ourselves, FadD and FadE are misannotated based on putative function compared with well-studied systems. The previous bioinformatics and annotations had predicted *S. aureus* lacked the bifunctional enzyme, FadB (a crotonase and hydroxyacyl-CoA dehydrogenase enzyme), which is essential to the Fad pathway (18, 48). However, new discoveries indicate that *S. aureus* encodes all the proteins necessary for a complete pathway. Here, we provide data demonstrating that the *S. aureus* FadB protein does possess both conserved domains necessary for its bifunctional activity (Fig. 6). Our own bioinformatic analysis revealed the enzymatic domains are in opposite orientation compared with the well-characterized *E. coli* FadB protein. This was further supported by overlaying the AlphaFold model of each SaFadB domain with that of the *E. coli* FadB and identified strong similarities (Fig. S7). In this study, we were able to complement single *E. coli fadA* and *fadB* mutants on various fatty acids with the *safadBA* genes provided on a plasmid. We also identified that the SaFadB domains possess the conserved amino acid residues essential for function. Although the hydroxyacyl-CoA dehydrogenase activity of *S. aureus* FadB was recently confirmed using purified protein (49), the crotonase activity has not been examined. In this study, we demonstrate for the first time that mutations of the conserved amino acids in the SaFadB crotonase domain resulted in the inability of the SaFadBA proteins to restore growth of an *E. coli* Δ*fadB* mutant on a fatty acid source (Fig. 6E). These data support a new emerging model where *S. aureus* can metabolize exoFAs through β-oxidation.

Bacteria often suppress the expression of metabolic pathways that are not necessary when a preferred carbon source is present. *S. aureus* prefers to convert glucose to acetate in rich media, then switches to other metabolic pathways upon glucose exhaustion. Since fatty acids are not a preferred carbon source, it is not surprising that *fad* would be under strong regulatory control. In gram-positive bacteria, CcpA is the main transcriptional regulator of carbon metabolism in the presence of glucose and other sugars entering glycolysis and represses secondary metabolism when these preferred carbon sources are present. Although a previous study noted that P*_fadX_* is expressed higher in the absence of glucose, the mechanism was not identified (17). Here, we show that P*_fadX_* is under CcpA repression. Several pieces of evidence support this. First, P*_fadX_* expression increases from 4 h to 6 h, time points under our conditions where glucose is present and absent, respectively (28). Second, P*_fadX_* expression is repressed by both glucose and fructose, two carbon sources known to exert control over CcpA. Finally, P*_fadX_* expression increases in a Δ*ccpA* mutant when glucose is present, but there is no difference in the absence of glucose compared with wild-type. Together, these data are consistent with CcpA regulation. It should also be noted that we observed increased expression in P_IR1_ when CcpA was absent. This could indicate CcpA activity in this intergenic region; however, the expression of a potential promoter in this region is very low, and the biological significance of this is uncertain.

Although our data support P*_fadX_* repression by CcpA, we also uncovered evidence of additional regulation. Although P*_fadX_* expression was higher in a Δ*ccpA* mutant when glucose was present compared with the wild type, it was synergistically derepressed in the absence of glucose and Δ*ccpA* (compare blue columns in Fig. 5B). This is hard to reconcile if CcpA is the only regulator of *fad* expression and alludes to the involvement of other regulatory proteins; therefore, we hypothesize that an additional regulator also acts on P*_fadX_*. Interestingly, the *fadX* promoter includes an inverted repeat (TTTACACaaaGTGTAAA) between the putative −10 and the gene. Whether this is a binding site of an unidentified regulatory protein is uncertain. Some bacteria, such as *E. coli*, possess a multifunctional regulator, FadR, that derepresses the β-oxidation genes in the presence of long-chain acyl-CoA molecules (14, 50–52). Currently, *S. aureus* is not known to possess a FadR regulator; however, it does possess another candidate, the transcriptional regulator FapR, that surveys the levels of malonyl-CoA and regulates the expression of genes needed for fatty acid biosynthesis (53–55). Further studies will be needed to identify additional regulatory proteins and define the regulation of Fad.

The Fad pathway could serve several purposes, such as aiding in membrane homeostasis during exoFA encounters, metabolizing fatty acids for energy, or truncation of exoFAs as part of the detoxification process. The question remains whether *S. aureus* performs fatty acid degradation. A single experiment suggested that [^14^C]oleic acid is not degraded by the *S. aureus* RN4220 strain (16). However, it is unknown whether unsaturated fatty acids, such as oleic acid, could serve as a substrate for the Fad pathway. Additionally, we have now shown the *fad* operon is under strong regulatory control, and expression of the operon was not determined in the previous study which used cells at OD_600_ of 1.3 and did not state whether media contained glucose (16). We confirmed that the first enzymatic step of the fatty acid degradation cycle, conversion of an acyl molecule to acyl-CoA, is dependent on the *fad* operon (Fig. S8) in agreement with previous findings (17). However, we were unable to detect other degradation products, including palmitic acid-derived [^13^C]acetyl-CoA in our wild-type strain (not shown). This was intriguing since we show the *fadXDEBA* genes are expressed similarly at the mRNA level. We also did not identify any promoters within IR1 or IR2, which could indicate distinct regulation of different *fad* genes and could account for the lack of full Fad activity detection in our samples. An analysis of RNA-seq data from our growth conditions and previously published 44 conditions (35), all indicate a similar expression pattern for the *fad* genes and do not support additional promoters within IR1 or IR2. Thus, this cannot account for the lack of identified Fad activity. Our current hypothesis is that the intergenic regions are locations of post-transcriptional regulation. This would account for the detection of the *fad* genes on a single mRNA but the lack of Fad activity. Whether this is the case or not will be the focus of future studies.

## MATERIALS AND METHODS

### Bacterial strains and growth conditions

For all experiments, *S. aureus* strains (Table 1) were cultured from −80°C freezer stocks on tryptic soy agar (TSA) and sub-cultured overnight in 3 mL of tryptic soy broth (TSB). TSB was supplemented with chloramphenicol (10 µg mL^−1^) when necessary. *E. coli* strains were grown in lysogeny broth (LB) medium supplemented with ampicillin (100 µg mL^−1^) when necessary. Unless otherwise stated, overnight cultures were diluted to OD_600_ = 0.1 in 12.5 mL of filter-sterilized TSB (with no dextrose, ThermoFisher cat#: 286210) and with 14 mM glucose when applicable) in 125 mL flasks.

**TABLE 1:** Bacterial strains and plasmids

Strain or plasmid	Description^*a*^	Reference(s) or source
Strains		
*Staphylococcus* *aureus*		
AH1263	USA300 CA-MRSA strain LAC without LAC-p03, wild-type strain used for these studies	(56)
JLB2	AH1263 Δ*fakA*	(57)
JLB113	AH1263 *ccpA*::tetL	(26)
JLB114	AH1263 Δ*fakA ccpA*::tetL	(28)
JLB333	AH1263 Δ*fadXDEBA*	This study
RN4220	Highly transformable *S. aureus*	(58)
*Escherichia coli*		
BW25113	Δ(*araD-araB*)*567,* Δ(*rhaD-rhaB*)*568, ΔlacZ4787* (::rrnB-3), *hsdR514, rph-1, λ^-^*	(59, 60)
CAG18496	MG1655 *fadA*751::Tn10, referred to as *fadA*::Tn	(61, 62)
DH5α	F^-^ 80d*lacZ*ΔM15, Δ(*lacZYA-argF, U169, deoR, supE44, hsdR17, recA1, endA1, gyrA96, thi-1, relA1*)	(63)
JW3822-1	BW25113 Δ*fadB*786::kan, referred to as Δ*fadB*	(59)
JW5578-1	BW25113 Δ*fadA*785::kan, referred to as Δ*fadA*	(59)
MG1655	*λ^-^*, *rph-1*	(64–66)
Plasmid		
pCM2	Translational reporter for *fadX* start codon "ATG"	This study
pCM3	Translational reporter for *fadX* start codon "TTG"	This study
pCM5	*fad* operon deletion plasmid	This study
pCM9	Translational reporter for the 1st IGR in *fad* operon	This study
pCM10	Translational reporter for the 2nd IGR in *fad* operon	This study
pCM25	*S. aureus fadB* mutations of E^550^A and E^570^A	This study
pCM31	*fadX*^Δ-35/-10^ translational reporter	This study
pZD16	*S. aureus fadBA* expression plasmid	This study
pZD20	*S. aureus fadA* expression plasmid	This study
pZD21	*S. aureus fadB* expression plasmid	This study
pCM28	*E. coli-S. aureus* shuttle vector, Amp/Cm^R^, ColE1 and pC194 origins	(67)
pJB38	Temperature-sensitive allelic exchange vector, Amp/Cm^R^, ColE1 and pE194ts origins	(68)
pJB165	*fakA* complement	(57)
pJB185	Promoterless codon-optimized *lacZ,* Amp/Cm^R^, ColE1 and pC194 origins	(69)
pDSW208	*E. coli* IPTG-inducible plasmid. Fusion vector	(70)

^*a*^ tetL and kan denote tetracycline and kanamycin-resistant cassettes, respectively. Amp^R^ and Cm^R^ denote ampicillin and chloramphenicol resistance cassettes.

### Cloning

PCR was performed using oligos synthesized by Integrated DNA Technologies using the KOD DNA Polymerase kit (Sigma Aldrich). Plasmids were isolated using the Promega Wizard *Plus* SV Minipreps DNA Purification System (Promega, Madison, WI). DNA digests and ligations were performed using enzymes from New England Biolabs and were cleaned with Zymo Research Clean and Concentrator kits between steps. Sequencing of inserts was performed by ACGT, Inc. Details of plasmid construction can be found in Table S1. pCM25 was generated by GenScript.

### β-galactosidase assays

Cultures were grown for 4 or 6 h at 37°C with shaking at 250 RPM. At each time point, 1 mL of culture was harvested, and cell pellets were stored at −80°C until the assay was performed. The cells were resuspended in 1.2 mL of Z-buffer and subjected to lysis using the FastPrep-24 5G homogenizer (MP Biomedicals) using 0.1 mm glass beads and the manufacturer’s setting for *S. aureus*. Cellular debris was pelleted by centrifugation at 21,130 × *g* for 5 min, and the lysate was transferred to a new tube. A sample of lysate was removed (50–200 µL) and combined with Z-buffer for a total volume of 700 µL to which 140 µL of *o*-nitrophenyl β-D-galactopyranoside (ONPG) (4 mg mL^−1^ [wt/vol, in 40 mM NaH_2_PO_4_, 60 mM Na_2_HPO_4_, pH 7.0]) was added and incubated statically at 37°C until the sample turned slightly yellow. At this point, 200 µL of 1 M NaCO_3_ was added to stop the reaction, and the samples were transferred to a cuvette and absorbance read at 420 nm. Bradford assays were performed by using the Protein Assay Dye Reagent (Bio-Rad, Hercules, CA). Miller units were calculated based on the micrograms of protein.

### Real-time PCR

Cultures were grown for 4 or 6 h (exponential phase), and 3 mL of culture was then transferred to a conical tube and centrifuged at 4,500 *× g* for 5 min at 4°C. The supernatant was removed, and the pellet was frozen at −80°C. After thawing on ice, the pellet was resuspended in TE buffer and transferred to a Lysing Matrix B Tube (MP Biomedicals), and the cells were subjected to lysis using the FastPrep-24 5G homogenizer (MP Biomedicals). Total RNA was extracted using the RNeasy Mini Kit (Qiagen) and treated with DNase (TURBO DNA-*free* Kit [Invitrogen]). RNA was quantified using a NanoDrop One (ThermoFisher Scientific), and cDNA was synthesized using the QuantiTect Reverse Transcription Kit (Qiagen) with 500 ng of total RNA used as the template. The cDNA was diluted 50-fold in nuclease-free H_2_O. A reaction mixture containing FastStart Essential DNA Green Master (Roche), primers (5 µM each, designated as RT-*fad*F for forward and RT-*fad*R for reverse), and H_2_O was added to the diluted cDNA. Finally, 19 µL of reaction mixture combined with cDNA was aliquoted in triplicate into a 96-well plate, and amplification was performed using a LightCycler96 (Roche). The calibrator for calculations was *sigA* (*rpoD*) (71), as it is a common calibrator used in our field (72–74), and we observed no significant difference in *rpoD* expression (i.e., C_T_ values) in any of the strains under these conditions.

### RNA-seq

RNA-seq libraries were created by the Ohio University Genomics Facility using rRNA-depleted RNA samples and the Illumina Truseq stranded mRNA library prep kit (75). Libraries were sequenced on a MiSeq Sequencing System. Data were exported and analyzed using CLC Genomics Workbench as described previously (76). Reads corresponding to rRNA were removed prior to analysis, and expression values were calculated for each gene using the RNA-seq function. The results were normalized using quantile normalization. Lowly expressed genes (i.e., where expression values were <10 in both conditions) were excluded, as were genes in which < 80% of the mapped reads were non-unique. The RNA-seq data are available from Gene Expression Omnibus under accession number GSE299037.

### SaFad complementation assay in *E. coli*

Overnight *E. coli* cultures grown in LB were centrifuged for 10 min at 4,500 × *g* and then washed with 3 mL 1× phosphate-buffered saline (PBS) and concentrated again for 10 min at 4,500 × *g*. The pellets were resuspended in 3 mL M9 media supplemented with 20% glucose and grown to exponential phase at 37°C with shaking at 250 RPM, and 2 mL of each culture was then concentrated, washed with M9 media with no carbon source, then resuspended in M9 no carbon source; 2 µL of each culture containing equal number of cells (OD_600_ = 5.0) were then spotted on M9 media plates supplemented with a single fatty acid and incubated at 25°C static for 7 days. Saturated fatty acids were dissolved in 100% ethanol or chloroform and then added to M9 agar media at a final concentration of 2 mM. Oleic acid was added directly from stock into the M9 agar media to a final concentration of 2 mM. At day 7, the plates were imaged and/or colonies were cut out and vortexed in 1× PBS. A dilution series was performed for each colony and track plated on TSA plates. CFUs were quantified following overnight incubation at 37°C static overnight.

### Reverse-transcription PCR

RNA was isolated from cells grown for 6 h. cDNA was synthesized from 500 ng total RNA using the QuantiTect Reverse Transcription Kit (Qiagen), a gene-specific primer (CM53 and CM56), and a 45-min synthesis step. A PCR was then performed using the KOD DNA Polymerase kit (Sigma Aldrich) and cDNA as the template using primers CM51 and CM52 for IR1 and CM54 and CM60 for IR2.

### Statistical analysis

Statistical analysis was performed using GraphPad Prism using the tests indicated in the figure legends.

## SUPPLEMENTAL MATERIAL

Supplemental figures and tables jb.00089-25-s0001.pdf Fig. S1 to S9, and Tables S1 to S2.

ASM does not own the copyrights to Supplemental Material that may be linked to, or accessed through, an article. The authors have granted ASM a non-exclusive, world-wide license to publish the Supplemental Material files. Please contact the corresponding author directly for reuse.
