# Supplementary material for: Characterizing the Staphylococcus aureus fatty acid degradation operon
Source: J Bacteriol. 2025 Jul 17;207(8):e00089-25. doi: 10.1128/jb.00089-25 (PMC12369327; doi:10.1128/jb.00089-25)
Supplement: Supplemental figures and tables — Fig. S1 to S9, and Tables S1 to S2. [file jb.00089-25-s0001.pdf]

Supplemental information for

Characterizing the *Staphylococcus aureus* fatty acid degradation operon

Cindy Menjivar[1], Zachary R. DeMars[1], Richard E. Wiemels[2], Ronan K. Carroll[2], Jeffrey L. Bose[1*]

[1]Department of Microbiology, Molecular Genetics, and Immunology, University of Kansas Medical Center, Kansas City, Kansas, USA

[2]Department of Biological Sciences, Ohio University, Athens, Ohio, USA

This file includes:

Figure S1. Schematic of *fadX* promoter and *fad* locus.
Figure S2. The *fad* genes respond similarly.
Figure S3. Additional *fad* expression information.
Figure S4. Growth curves of strains.
Figure S5. Putative *cre* sites in the *fad* locus.
Figure S6. Representative images of additional plate growth conditions.
Figure S7. Modeling of *S. aureus* FadB.
Figure S8. Detection of Fad intermediates.
Figure S9. Full gel images.

Table S1. Cloning strategy
Table S2. Oligonucleotides

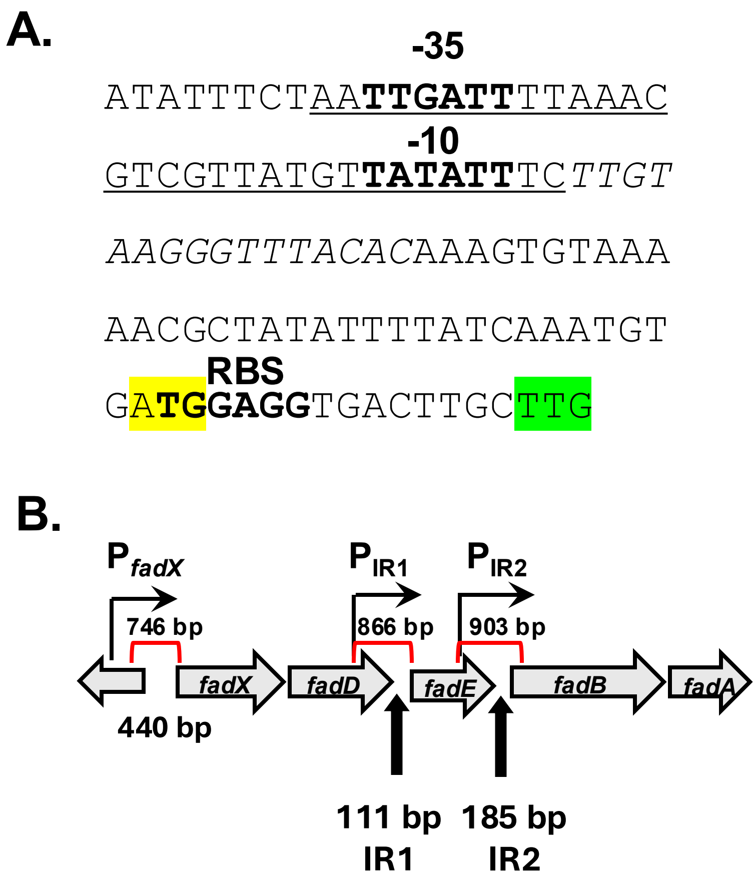

Figure S1. Schematic of *fadX* promoter and *fad* locus. A) Schematic of *fadX* promoter elements including putative -35/-10 (bolded) based on the consensus sequence of TTGACA and TATAAT, putative ribosomal binding site (RBS, bolded), the identified translational start site (highlighted in green), and the annotated translational start site (highlighted in yellow). Also indicated are the deleted sequence for the -35/-10 β-galactosidase reporter plasmid (underlined) and the putative *cre* site (italics). **B)** Diagram of the cloning regions for β-galactosidase reporters P_{fadX}, P_{IR1}, P_{IR2}.

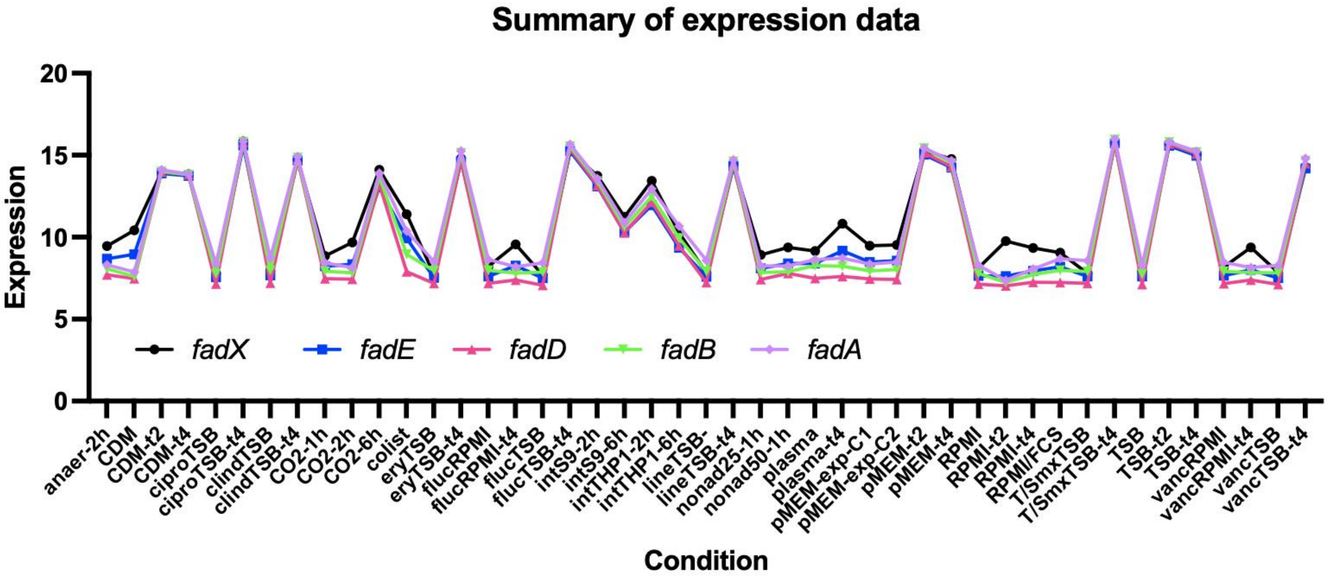

Figure S2. The *fad* genes respond similarly. RNAseq data was analyzed from Mader 2016 and plotted for each *fad* gene.

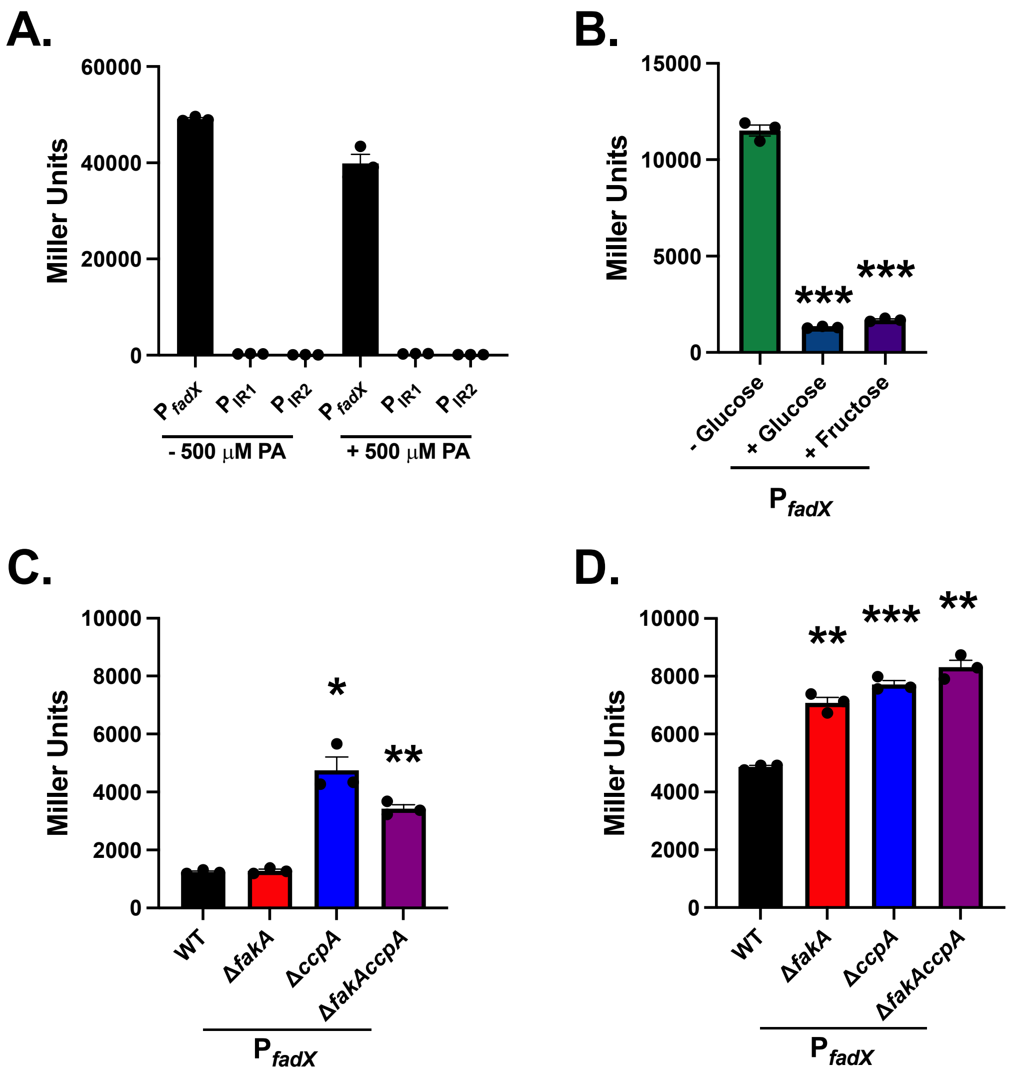

Figure S3. Additional *fad* expression information. A) β-galactosidase assay with wild-type strain harboring reporter plasmids for P_{fadX} and the two intergenic regions (P_{IR1} and P_{IR2}) grown for 8 hours in TSB no glucose with or without 500 µM palmitic acid (PA). **B)** β-galactosidase assay with wild-type strain harboring reporter plasmids for P_{fadX} grown for 4 hours in TSB no glucose and TSB no glucose supplemented with 14 mM glucose or 14 mM fructose. **C and D)** β-galactosidase assay with wild-type (WT) strain, Δ*fakA,* Δ*ccpA,* and Δ*fakA* Δ*ccpA* mutants harboring the P_{fadX}-*lacZ* reporter plasmid and grown to **C)** 4 hours and **D)** 6 hours in TSB supplemented with 14mM glucose. Bars represent the mean (n=3) with SEM. * indicates p<0.05, ** indicates p<0.01, *** indicates p<0.001 as compared to wild-type by student's t-test.

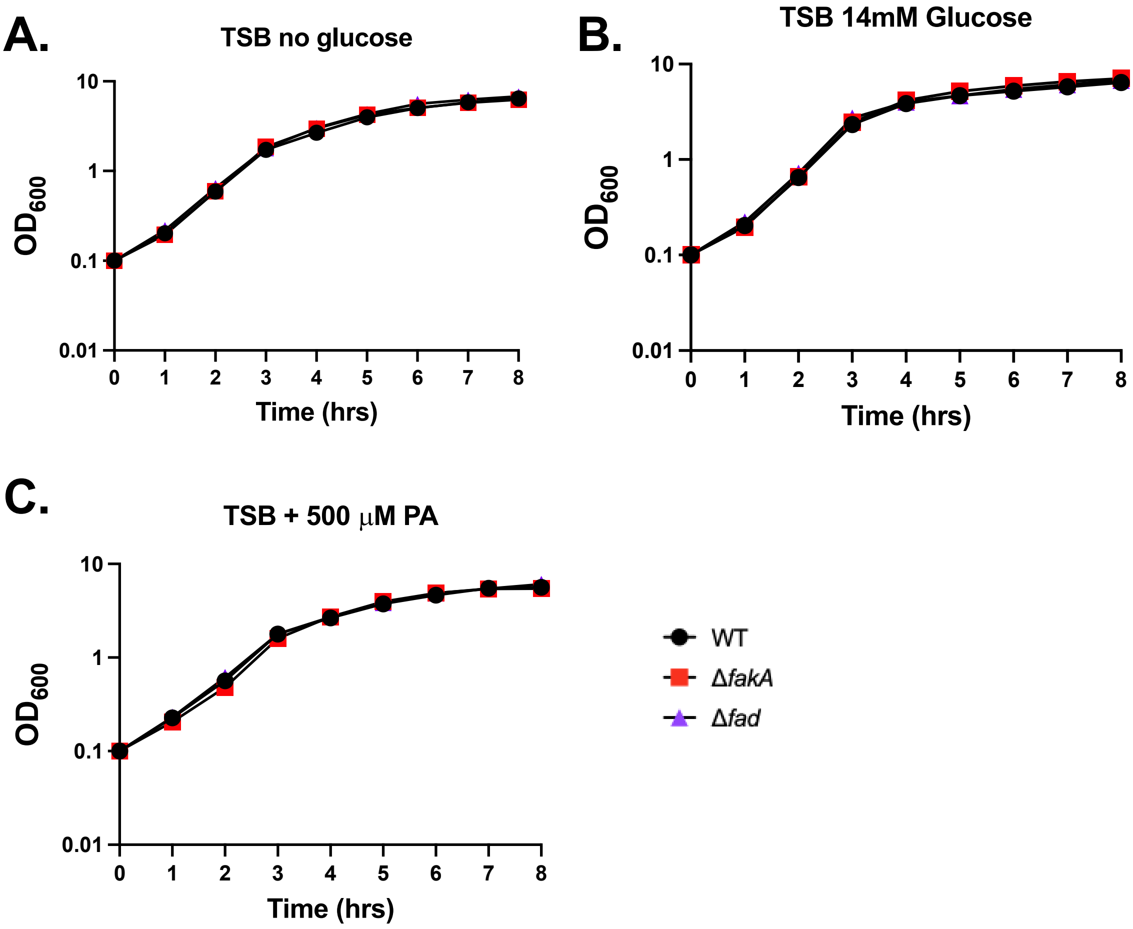

Figure S4. Growth curves of strains. Wild type (WT), Δ*fakA,* and Δ*fadXDEBA* (Δ*fad*) mutant were grown 8 hours at a 1:10 media to flask ratio in **A)** TSB without glucose, **B)** TSB supplemented with 14 mM glucose, and **C)** TSB supplemented with 500 µM palmitic acid (PA). Samples were taken every hour. Symbols represent the mean (n=3) with SEM. Error bars are present and may be smaller than symbols.

A.

```
Consensus:    WTGNAARCGNWNNCAW
Site 1        TTGTAAGGGTTTACAC
Site 2        TTGTAAACGCTTACAC
B. subtilis    TGAAANCGNTTNCA
GAS           WWGAAANCGNTTNCA
```

B.

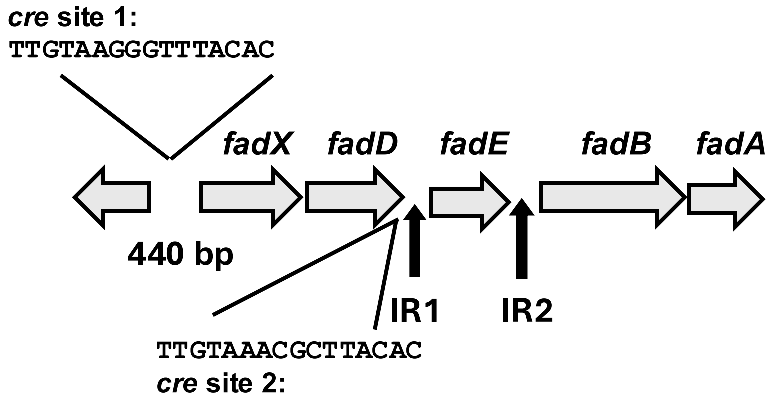

Figure S5. Putative *cre* sites in the *fad* locus. A) Consensus sequence for *cre* site in comparison to identified Fad site 1 and site 2 as well as *Bacillus subtilis* and Group A *Streptococcus* (GAS). Where W= A or T, R= G or A, N= any nucleotide. **B)** Schematic of the *S. aureus fadXDEBA* operon with putative *cre* sites indicated. Sequences in panel A are based on (DebRoy, 2020 and Marciniak, 2012).

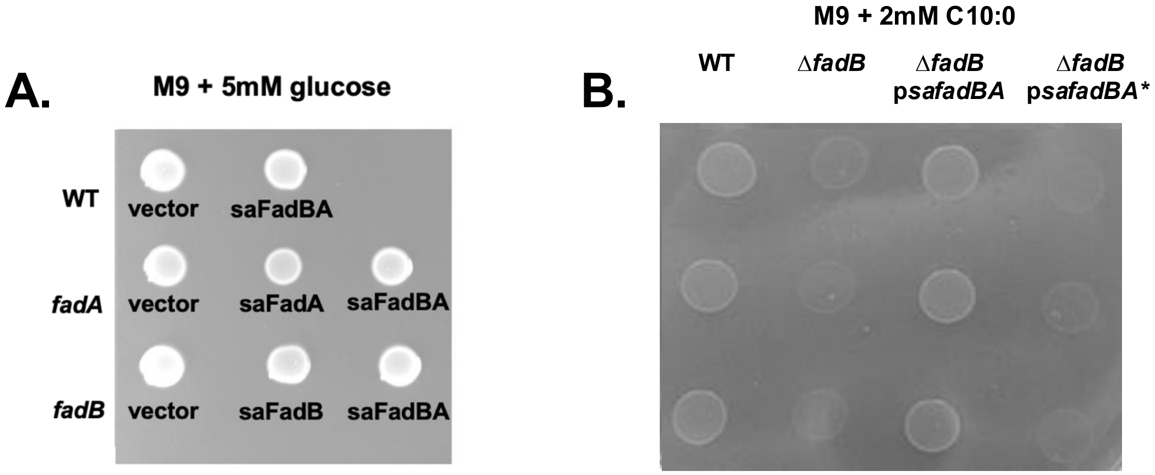

Figure S6. Representative images of additional plate growth conditions. A) Growth of strains on minimal media containing 5 mM glucose, 0.1mM IPTG, and 100 μg ml^{-1} ampicillin. Top row: MG1655 *E. coli* wild-type (WT) harboring empty vector or SaFadBA. Middle row: *fadA*::Tn10 harboring empty vector or SaFadBA and Δ*fadA* harboring plasmid expressing SaFadA. Bottom row: Δ*fadB* mutant harboring empty vector or plasmid expressing SaFadB or SaFadBA. Image is representative of several independent experiments. **B)** Growth of SaFadB crotonase mutant on minimal media plates containing 2 mM C10:0. Equal numbers of *E. coli* BW25113 wild-type (WT), Δ*fadB* mutant, and mutant harboring plasmids expressing SaFadBA or SaFadBA* (E550A and E570A in FadB) were grown in triplicates and grown for 7 days.

A.

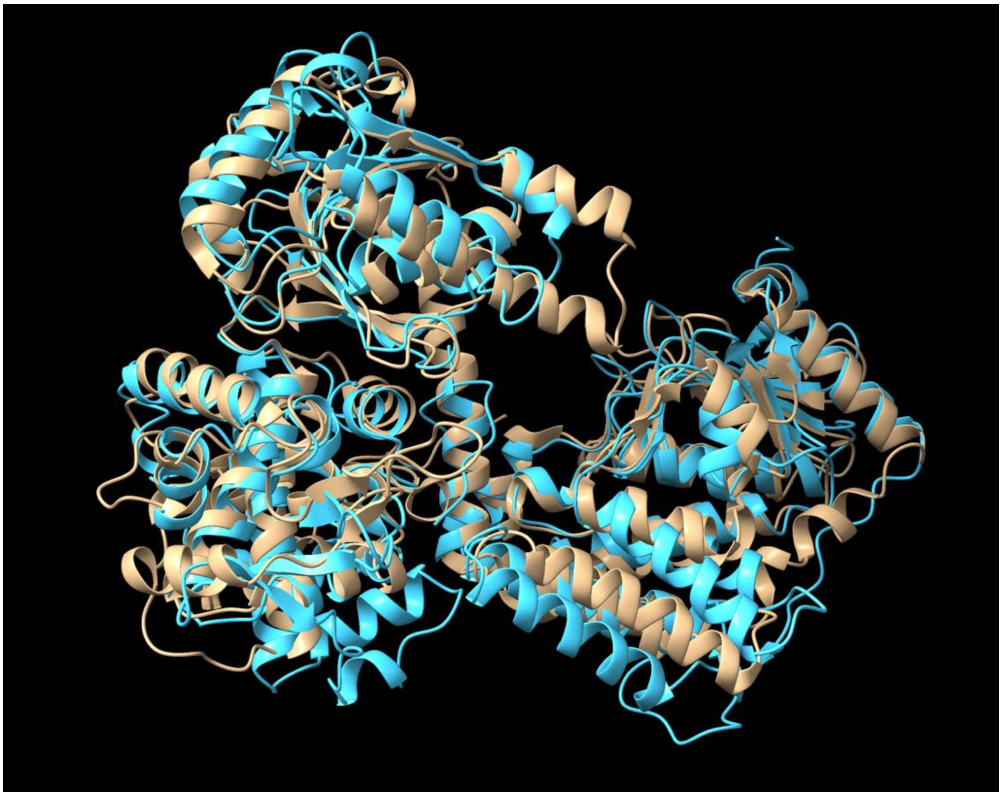

B.

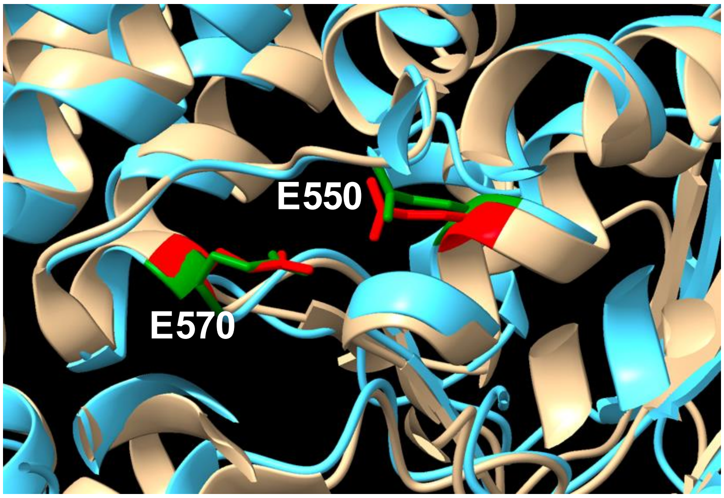

Figure S7. **Modeling of *S. aureus* FadB. A)** Overlay of *E. coli* FadB (PDB: 6tnm, blue) and AlphaFold model of *S. aureus* FadB (tan). The *S. aureus* FadB was split into N-term (amino acids 1-422) and C-term (423-753) before matchmaking in ChimeraX. **B)** Zoomed in area around active site of the crotonase domain with the catalytic glutamate residues in red (*E. coli*) and green (*S. aureus*). Number labels are based on the *S. aureus* amino acid sequence.

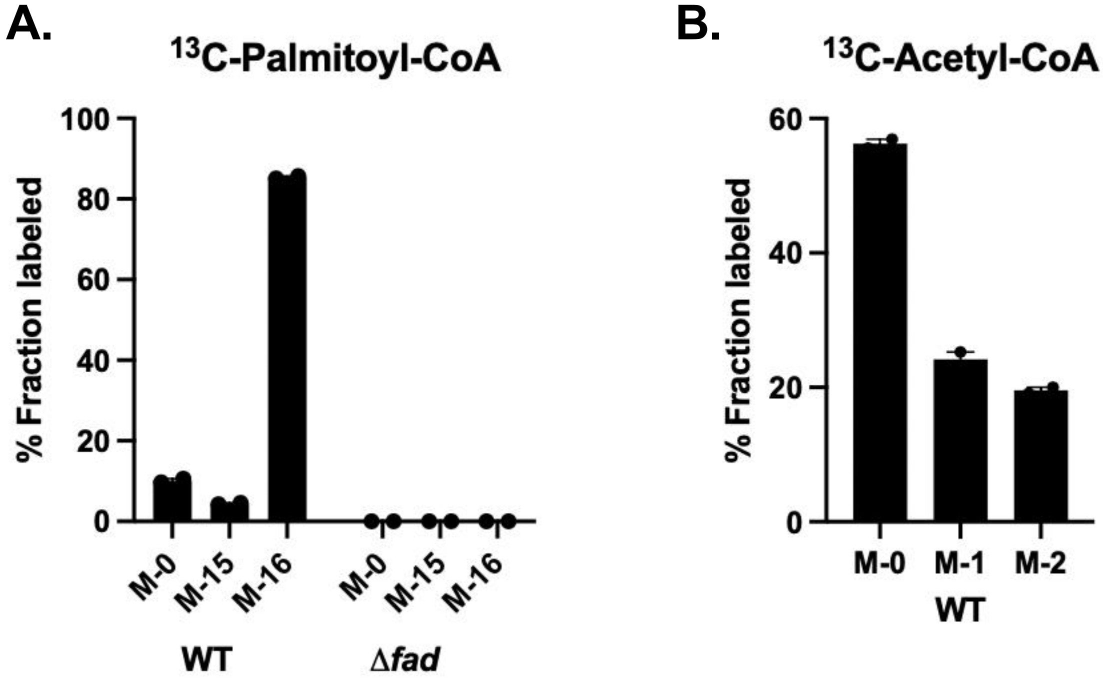

Figure S8. Detection of Fad intermediates. A) The wild-type (WT) strain and the Δ*fadXDEBA* (Δ*fad*) mutant were grown in TSB without glucose for 4 hours total, with 500 μM [^{13}C]palmitic acid added after 2 hours of growth. Cell pellets were analyzed by mass spectrometry. M-0, M-15, and M-16 indicates fraction of palmitoyl-CoA in the cell with no label, 15 carbons or 16 carbons with the label, respectively. **B)** Control experiment of WT grown in TSB supplemented with 14mM [^{13}C]glucose. M-0, M-1, and M-2 indicates fraction of acetyl-CoA in the cell with no label, 1 carbon or 2 carbons with the label, respectively.

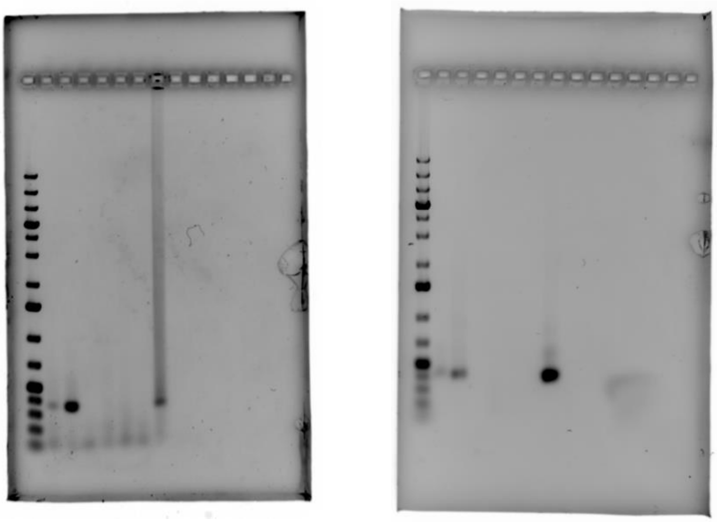

Figure S9. Full gel images. Uncropped images used to produce Figure 4. lanes 10-15 are empty.

Supplemental Table 1. Cloning

Plasmids	Cloning strategy	Reference or source
pCM1	Upstream deletion for *fadXDEBA* operon (primers CM1 and CM5, AH1263 template). Cloned into EcoR1 and Kpn1 sites of pJB38.	This study
pCM2	Translational reporter for *fadX* start codon "ATG" (primers CM1 and CM7, AH1263 template). Cloned into EcoR1 and Xba1 sites of pJB185.	This study
pCM3	Translational reporter for *fadX* start codon "TTG" (primers CM1 and CM8, AH1263 template). Cloned into EcoR1 and Xba1 sites of pJB185.	This study
pCM5	*fad* operon deletion plasmid (primers CM9 and CM10 were used for downstream of the *fad* operon, AH1263 template). Sequentially cloned into Kpn1 and Sal1 sites of pCM1.	This study
pCM9	Translational reporter for the 1st IGR in *fad* operon (primers CM11 and CM25, AH1263 as template). Cloned into EcoR1 and Xba1 sites of pJB185.	This study
pCM10	Translational reporter for the 2nd IGR in the *fad* operon (primers CM15 and CM26, AH1263 as template). Cloned into EcoR1 and Xba1 sites of pJB185.	This study
pCM25	Ordered from GenScript. *fadB* mutations of $E^{550}A$ and $E^{570}A$. Used pZD16 as vector.	This study
pCM27	For *fadX*$^{\Delta\text{-}35/\text{-}10}$ translational reporter. Upstream of the -35 sequence in *fadX* promoter region (primers CM1 and CM68, pCM3 as template) and cloned into EcoRI and Nhe1 sites of pCM28.	This study
pCM31	For *fadX*$^{\Delta\text{-}35/\text{-}10}$ translational reporter. Downstream of -10 sequence in f*adX* promoter region and includes *lacZ* gene (primers CM69 and LacZR with pCM3 as template) and cloned into Nhe1 and Pst1 sites of pCM27.	This study
pZD15	*E. coli* IPTG-inducible plasmid. Removed GFP from pDSW208 vector (primers ZD26 and ZD27), Amp^R	This study
pZD16	*S. aureus fadBA* expression plasmid. Has native ribosome binding site (primers ZD23 and ZD25 with AH1263 as template). Cloned into BamH1 and Sal1 sites of pZD15.	This study
pZD20	*S. aureus fadA* expression plasmid. Has native ribosome binding site (primers ZD40 and ZD41 with AH1263 as template). Cloned into EcoR1 and Sal1 sites of pZD15.	This study
pZD21	*S. aureus fadB* expression plasmid. Has native ribosome binding site (primers ZD23 and ZD39 with AH1263 as template). Cloned into BamH1 and Sal1 sites of pZD15.	This study
pJB38	Temperature-sensitive allelic exchange vector, Amp/Cm^R, ColE1 and pE194ts origins	Bose, 2013

pJB185	Promoterless codon-optimized *lacZ,* Amp/Cm^R, ColE1 and pC194 origins	Krute, 2017
pCM28	*E. coli-S. aureus* shuttle vector, Amp/Cm^R, ColE1 and pC194 origins	Pang, 2010
pDSW208	*E. coli* IPTG-inducible plasmid. Fusion vector.	Weiss, 1999
pJB165	*fakA* complement	Bose, 2014

Supplemental Table 2. Oligonucleotides

**Oligonucleotides**[a]	**Sequence (5'-3')**[b]	**Reference or source**
CM1	ccgaattCACAAATTGCTGGCGTAGTTTTAGC	This study
CM5	caggtaccCATCACATTTGATAAAATATAGCGTTTTTACAC	This study
CM7	cgtctagaCATCACATTTGATAAAATATAGCGTTTTTACAC	This study
CM8	cctctagaCAAGCAAGTCACCTCCATCACATTTGATAA	This study
CM9	tgggtaccTAGAATGGTTGATTTTGGATGAAGC	This study
CM10	caggtcgacGTTTGTAACTCTATCCAAAGACATACAGTC	This study
CM11	gtcgaattcCGCTACGTGCTAGTAATTTTAATCCTG	This study
CM15	gtcgaattcAGGCGATACTTGGGTTATCAATGG	This study
CM25	gatctagaTGTCATATAAATTCCTCCTAAAAATAATATG	This study
CM26	catctagaCATAGTGATTCCTCCAATTTAGTTGAGGATAAG	This study
CM49<br>RT-*fadB*F	AAGGTGATGCCAAACTTTCC	This study
CM50<br>RT-*fadB*R	ATCGCATCAACTAACGCATC	This study
RT-*fadX*F	GCCTTGTCTGGAGAAGAACG	This study
RT-*fadX*R	GTTCACGACTCCCAACGAAT	This study
CM72<br>RT-*fadD*F	GTTCGTTGATGCACGTATCC	This study
CM73<br>RT-*fadD*R	TGAAATGCTTTGGCAGTCTC	This study
RT-*fadE*F	CCAAGGCGATACTTGGGTTA	This study
RT-*fadE*R	TAGGGCGTTAGGAACAATGC	This study
RT-*fadA*F	TGCATATGCGATTCCTGAAG	This study
RT-*fadA*R	TAACATTGCGCCTGTAGCAC	This study
CM51	CATATGCGAAGTCGTTTAGCAGGTTATAAAGTCCCAAG	This study
CM52	ATTCGACTTCACCGTCTGTTAAACCTTTAGCAATAC	This study
CM53	GTCCTCGGGTACAGTAGCGTCTATCCAATG	This study
CM54	GATGCAGAAGCGATTTACACGTACGAAGGTACAC	This study
CM56	AGTCGAATAGTAGCGGCCGTTTCTTATCTGTA	This study
CM60	CAATGAGATTTGGATCGTTTTGTCCACTAC	This study
CM68	cagctagcGAAATATCTTTTTATTCTGATAATAGACACAG	This study
CM69	cagctagcCTTGTAAGGGTTTACACAAAGTGTAAAAACGC	This study

CMLacZR*	GCTTGCATGCCTGCAGTTATTTTTGAC	This study
CMLacZ1*	GATTCATTAGCAGTTGTTTTACAACGTCGTGATTG	This study
CMLacZ2*	CAGATGGTTCATATTTAGAAGATCAAGATATGTG	This study
CMLacZ3*	GATCCATCACGTCCAGTGCAATATGAAGGTG	This study
CM79*	GACACTGAATTTGCTCAAATTTTTGTTTGTAGAATTAG A	This study
JBCM28F*	acgcaattaatgtgagttagctcactcattag	This study
ZD23	tatggatccCTCAACTAAATTGGAGGAATCACTATG	This study
ZD25	agcgtcgacCTAACGCACATATTCAAATATAG	This study
ZD26	gtttgacagcttatcatcgactgcacg	This study
ZD27	AAAGAGTTTGTAGAAACGCAAAAAGGCCATC	This study
ZD28*	TTGTCGTCAATGATGTGCCTGGCTTTG	This study
ZD29*	GTTAAAACACGTATGGAAGACGAACTTGG	This study
ZD30*	TTGGCTTATTACCGAGTGGCGGTGG	This study
ZD31*	TGAAAGAATTGCACATATGTTAAAAACT	This study
ZD32*	TCCCAATTTGACGTATCACGTGAAGAT	This study
ZD40	aatgaattcAGTCATTAAGAGAGGATGATAACCATGC	This study
ZD41	aagtcgacACAAAACGAATCCGCTTCAT	This study
ZD39	acagtcgacTTATCATCCTCTCTTAATGACTATCTTTTA	This study
ZD32	TCCCAATTTGACGTATCACGTGAAGAT	This study
JBSIGAF*	AACTGAATCCAAGTGATCTTAGTG	Lehman, 2015
JBSIGAR*	TCATCACCTTGTTCAATACGTTTG	Lehman, 2015

[a] RT denotes primers used for real-time PCR

[b] lower-case bases denote those added for cloning purposes

* Denotes primers used for sequencing confirmation.
